# 938. Development of a Multidisciplinary Rounding Checklist to Optimize Geriatric Burn Care

**DOI:** 10.1093/jbcr/irag033.505

**Published:** 2026-04-06

**Authors:** Drashti Patel, Amalia Cochran, Lorraine A Todor, Anthony DePiero, Jillian K Furlong, Stephanie Yakoubovitch, Andrea Munden

**Affiliations:** University of Florida, Gainesville, FL; University of Florida, Gainesville, FL; Tampa General Hospital, Tampa, FL; University of Florida Health, Gainesville, FL; University of Florida Health, Gainesville, FL; University of Florida Health, Gainesville, FL; University of Florida, Gainesville, FL

## Abstract

**Introduction:**

Geriatric burn patients represent a growing and vulnerable population, accounting for nearly one-third of adult admissions at our regional American Burn Association verified burn center. Patients over the age of 65 experience higher morbidity and mortality during hospitalization, and a multidisciplinary approach to care can help mitigate complications. This study describes the process of integrating multidisciplinary recommendations into a patient care checklist suitable for use during daily rounds, with the goal of improving care for geriatric burn patients.

**Methods:**

Beginning in April 2025, an interdisciplinary team began a series of monthly meetings designed to identify areas for improvement in the care of geriatric patients. Representation included occupational therapy (OT), physical therapy (PT), speech-language pathology (SLP), nutrition, pharmacy, and geriatric medicine. Using an iterative review and feedback process from all team members, an interdisciplinary checklist was developed to ensure application of best care principles for all geriatric burn patients. This work was IRB exempt as a process improvement project.

**Results:**

Each team member provided recommendations for best practices within their discipline. Over subsequent meetings, these recommendations were reviewed and refined to develop consensus; one of the group’s goals was to ensure best care in a way that would not become onerous for any member of the care team. Recommendations from the interdisciplinary team ultimately yielded a standardized rounding checklist (Fig. 1). Baseline mobility, swallowing function, and nutrition were systematically evaluated to prevent deconditioning. Delirium prevention was protocolized, and medication reconciliation addressed polypharmacy and opportunities for de-prescribing. Student volunteers and arts in medicine therapy were recruited for psychosocial support during lengthy hospitalizations.

**Conclusions:**

Standardized rounding checklists can be systematically developed and integrated in burn centers to enhance interprofessional collaboration and improve adherence to geriatric best practice guidelines. The next phase of this project will involve the implementation of the developed checklist to examine if we are able to improve prognostication and reduce adverse outcomes within this vulnerable patient population.

**Applicability of Research to Practice:**

Standardized checklists are a feasible quality improvement effort, ensuring that each member of an interdisciplinary team is appropriately included in the patient’s care and that best practices are routinely applied. Integration into electronic health records and ongoing staff education can improve efficiency and sustainability.

**Funding for the study:**

N/A.

